# Enhanced U-Net with GridMask (EUGNet): A Novel Approach for Robotic Surgical Tool Segmentation

**DOI:** 10.3390/jimaging9120282

**Published:** 2023-12-18

**Authors:** Mostafa Daneshgar Rahbar, Seyed Ziae Mousavi Mojab

**Affiliations:** 1 Department of Electrical and Computer Engineering, Lawrence Technological University, Southfield, MI 48075, USA; 2 Department of Computer Science, Wayne State University, Detroit, MI 48202, USA; mousavi@wayne.edu

**Keywords:** minimally invasive surgery, convolutional neural network, U-Net, data augmentation, surgical tools segmentation, computer vision, image processing

## Abstract

This study proposed enhanced U-Net with GridMask (EUGNet) image augmentation techniques focused on pixel manipulation, emphasizing GridMask augmentation. This study introduces EUGNet, which incorporates GridMask augmentation to address U-Net’s limitations. EUGNet features a deep contextual encoder, residual connections, class-balancing loss, adaptive feature fusion, GridMask augmentation module, efficient implementation, and multi-modal fusion. These innovations enhance segmentation accuracy and robustness, making it well-suited for medical image analysis. The GridMask algorithm is detailed, demonstrating its distinct approach to pixel elimination, enhancing model adaptability to occlusions and local features. A comprehensive dataset of robotic surgical scenarios and instruments is used for evaluation, showcasing the framework’s robustness. Specifically, there are improvements of 1.6 percentage points in balanced accuracy for the foreground, 1.7 points in intersection over union (IoU), and 1.7 points in mean Dice similarity coefficient (DSC). These improvements are highly significant and have a substantial impact on inference speed. The inference speed, which is a critical factor in real-time applications, has seen a noteworthy reduction. It decreased from 0.163 milliseconds for the U-Net without GridMask to 0.097 milliseconds for the U-Net with GridMask.

## 1. Introduction

Minimally invasive surgery (MIS), often referred to as laparoscopic surgery or minimally invasive procedures, offers several notable advantages over traditional open surgery. These advantages include smaller incisions, resulting in less visible scarring [1], quicker patient recovery times, and shorter hospital stays [2]. MIS is associated with reduced postoperative pain and a decreased need for pain medication [3]. Additionally, the smaller incisions reduce the risk of surgical site infections, as there is less exposure to external contaminants [4]. Moreover, MIS procedures lead to reduced intraoperative and postoperative blood loss, which is particularly advantageous in cases where blood conservation is critical [5]. The minimized incision size also reduces the risk of wound complications, such as dehiscence, hernias, and evisceration [6], and results in better cosmetic outcomes [7]. Furthermore, MIS often enables patients to return to a regular diet more quickly, promoting early recovery, and can result in cost savings for both patients and healthcare systems [8].

MIS involves performing surgical procedures through small incisions rather than a large open incision. The surgical process begins with the surgeon creating several small incisions, typically ranging from 0.5 to 1.5 cm in length, near the surgical site [9]. These incisions serve as entry points for specialized surgical instruments and a camera. Subsequently, trocars, long, slender instruments, are inserted through these small incisions, providing access for the surgical instruments and the camera [10]. A laparoscope, a thin tube equipped with a camera and light source at its tip, is inserted through one of the trocars, providing high-definition images of the surgical area displayed on an operating room monitor. This real-time visual feedback guides the surgeon throughout the procedure. Specialized surgical instruments, such as scissors, graspers, cautery devices, and suturing tools, are inserted through the other trocars and are designed for various surgical tasks, such as cutting, cauterizing, and suturing. The surgeon manipulates these instruments from outside the body while monitoring the live video feed on the monitor, allowing for precise control and fine movements of the instruments [11]. Depending on the specific surgical procedure, the surgeon may manipulate tissues, remove or repair damaged structures, and complete the necessary steps to address the medical condition. Throughout the surgery, the surgical team continuously monitors the patient’s vital signs, such as heart rate, blood pressure, and oxygen saturation, to ensure the patient’s safety and well-being. After completing the surgical procedure, the surgeon removes the surgical instruments and trocars. The small incisions may be closed using sutures, surgical glue, or adhesive strips, or, in some cases, they may not require closure at all. The patient is then transferred to a recovery area, where they are monitored as they wake up from anesthesia. MIS finds applications in various medical specialties, including general surgery, gynecology, urology, orthopedics, and more [12].

While MIS offers numerous benefits, it also presents several challenges stemming from the intricate nature of the surgical procedure, limited field of view, complex hand-eye coordination requirements, and the involvement of human assistants. These challenges can lead to increased surgical time and costs. The complexity of performing intricate procedures through small incisions, sometimes with limited tactile feedback compared to open surgery, can be especially challenging when dealing with delicate anatomical structures. The use of small incisions and an endoscope reduces the surgeon’s field of view compared to open surgery, making it challenging to navigate and manipulate tissues effectively [13]. MIS necessitates that surgeons develop and maintain complex hand-eye coordination skills, translating external movements into precise actions within the body, often using instruments with articulating tips [14]. In many MIS procedures, a human assistant operates the endoscope’s camera, providing the surgeon with visual feedback, which introduces an element of dependency on the assistant’s skills and can affect surgery efficiency [15]. To address these challenges, there has been a noticeable surge of interest in the research domain of computer- and robot-assisted minimally invasive surgery (RAMIS) over the past few years. This heightened focus aims to enhance the capabilities of minimally invasive procedures, mitigate associated difficulties, and further improve patient outcomes, with researchers, clinicians, and healthcare institutions investing in exploring RAMIS’s potential benefits and advancements [16,17].

Advancements in this domain revolve around providing surgeons with effective tools to address and mitigate these challenges and open up exciting possibilities in surgical skill assessment, workflow optimization, and training of junior surgeons. One prominent approach involves harnessing information related to the positions and movements of surgical instruments during a procedure, often utilizing innovative tracking methods that enable real-time monitoring of surgical tools. These tracking methods, relying on technologies such as electromagnetic- or infrared-based systems or the strategic attachment of external markers to instruments, precisely capture instrument locations and movements within the surgical field, enhancing the surgeon’s capabilities and offering insights into surgical practice [18,19,20,21]. Presently, the primary focus in this domain is on utilizing visually derived data from endoscopic video streams, aligning with the advancements in deep-learning-based techniques within image processing [22,23].

The task at hand involves identifying and tracking surgical instruments within the surgical field, typically accomplished using object detection methods that locate instruments by enclosing them within bounding boxes. Once instruments are initially detected, tracking methods follow them across multiple video frames, ensuring consistent monitoring and recording of instrument positions and movements [24,25]. While these methods offer quick processing times, they may lack precision, particularly for instruments that extend from the bottom corners toward the center of the image. A significant advancement in surgical instrument detection and localization is achieved through segmentation methods, which provide a finer level of detail by predicting instrument shapes pixel by pixel. Unlike bounding box methods, segmentation offers a more accurate representation of instrument contours and boundaries [26,27,28,29]. A range of studies have explored surgical tool segmentation on the Johns Hopkins University (JHU) and Intuitive Surgical, Inc. (Sunnyvale, CA, USA. ISI) Gesture and Skill Assessment Working Set, JIGSAWS dataset, for autonomous image-based skill assessment. Papp [30] achieved promising results using TernausNet-11, while Ahmidi [31] reported high accuracy for gesture recognition techniques. Funke [32] demonstrated the feasibility of deep-learning-based skill assessment using 3D convolutional networks (ConvNets), and Lajkó [33] proposed a 2D image-based skill assessment method. Nema [34] and Jin [35] discussed the use of instrument detection and tracking technologies, with Jin introducing a new dataset and method for tool presence detection and spatial localization. Attia [36] achieved high accuracy in surgical tool segmentation using a hybrid deep convolutional neural network–recurrent neural network (CNN-RNN) auto-encoder–decoder. These studies collectively highlight the potential of various techniques for surgical tool segmentation and skill assessment on the JIGSAWS dataset.

This paper focuses on the application of the U-Net architecture, a CNN structure originally designed for precise pixel-wise image classification in medical image analysis tasks [26,27,28,37,38]. TernausNet, a U-Net architecture with a VGG11 pre-trained encoder, has shown superior performance in image segmentation tasks, particularly in the medical and satellite imaging domain [39]. This approach has been further extended to TernausNetV2, which allows for instance segmentation in high-resolution satellite imagery. The U-Net architecture, in general, has been widely adopted in medical image analysis, with various modifications and improvements proposed. These include the double U-Net, which combines two U-Net architectures and has shown improved performance in medical image segmentation [40], and UNet++, a nested U-Net architecture that has achieved significant gains in medical image segmentation tasks [41]. The U-NetPlus, a modified U-Net architecture with a pre-trained encoder, has also demonstrated superior performance in semantic and instance segmentation of surgical instruments from laparoscopic images [42]. The U-Net architecture’s potential is further enhanced by the integration of residual skip connections and recurrent feedback with a pre-trained EfficientNet encoder, resulting in improved segmentation performance [43]. However, the U-Net architecture has some common limitations, including limited receptive field, susceptibility to overfitting, challenges with imbalanced class distributions, difficulty handling irregular shapes, computational complexity, and issues with adapting to multiple data modalities [44,45,46,47,48,49]. To address these limitations and increase segmentation accuracy for surgical instruments, this paper proposes the integration of advanced augmentation techniques like MixUp, CutMix, or GridMask, with a specific focus on the GridMask technique.

In summary, MIS offers numerous advantages but presents unique challenges, leading to increased interest in computer RAMIS. Advancements in this field aim to enhance surgical capabilities, optimize workflows, and train junior surgeons. Tracking and real-time monitoring of surgical instruments play a crucial role in addressing these challenges, with segmentation methods offering higher precision. This paper focuses on the U-Net architecture’s application to surgical instrument segmentation, aiming to overcome its limitations by incorporating advanced augmentation techniques like GridMask.

## 2. Materials and Methods

Image augmentation techniques that rely on image erasure typically involve the removal of one or more specific portions within an image. The fundamental concept behind this approach is to substitute the pixel values in these removed regions with either constant or randomized values. In a study by DeVries and Taylor in 2017, they introduced a straightforward regularization method called “cutout.” This method entails randomly masking square regions within input data during the training of convolutional neural networks (CNNs). Cutout has been shown to enhance the resilience and overall performance of CNNs [50]. Another technique, proposed by Singh and others in 2018, is known as “Hide-and-Seek” (HaS). HaS involves randomly concealing patches within training images, encouraging the network to seek out other relevant information when crucial content is hidden [51]. In 2020, Zhong and his colleagues introduced the “random erasing” method. This technique randomly selects a rectangular area within an image and replaces its pixel values with random data. Despite its simplicity, this approach has demonstrated significant performance improvements [52]. A more recent development, presented by Chen et al. in 2020, delves into the necessity of information reduction and introduces a structured method called “GridMask”. GridMask is also based on the removal of regions within input images [53]. Unlike Cutout and HaS, GridMask does not eliminate continuous regions or randomly select squares; instead, it removes uniformly distributed square regions, allowing for control over their density and size. To address the trade-off between object occlusion and information retention, FenceMask, as proposed by Li et al. in 2020, employs a strategy simulating object occlusion. This method adds to the array of techniques based on the deletion of specific regions within input images [54].

GridMask is a data augmentation technique designed to enhance the performance of deep learning models, particularly in computer vision tasks [53]. The method involves overlaying a grid on the image and masking (or dropping out) certain regions, forcing the model to learn from a partial view of the data. The idea is akin to the way dropout works for neurons but is applied spatially on input images. Just like dropout prevents neurons from co-adapting and helps in regularization, GridMask ensures that the model does not overly rely on specific local features or pixels of the input. By dropping out certain sections of the image, the network is forced to learn more robust and generalized features. By masking out portions of the image, the model is pushed to use the surrounding context to make predictions about the masked regions. This is especially useful for segmentation tasks where understanding the context can be crucial. In real-world scenarios, the objects or regions of interest in images may be partially occluded. GridMask trains the model to handle such occlusions, making it more robust. U-Net, with its large number of parameters, can be prone to overfitting, especially when the dataset is limited. Data augmentation techniques like GridMask can effectively increase the size of the training dataset by providing varied versions of the same image, helping to reduce overfitting. GridMask forces the U-Net to focus on both local and global features. While the local features within the unmasked regions become more pronounced, the model also tries to infer global context from the available parts of the image. Data augmentation techniques often help in better convergence during training. By providing more varied data, GridMask can potentially smoothen the loss landscape and assist in more stable training. GridMask allows for random rotations, resizing, and shifting of the grid, leading to a wide range of augmentations from a single image. This ensures that the network encounters varied challenges during training, pushing it to learn a broader set of features. Incorporating GridMask into the U-Net training pipeline can be straightforward. It is essential to ensure that the augmentations are applied consistently to both input images and their corresponding masks/annotations during training, especially for segmentation tasks. As with any augmentation technique, it is advisable to monitor validation performance to ensure the augmentations lead to genuine improvements and not just make the task harder without yielding benefits.

### 2.1. Enhanced U-Net with GridMask (EUGNet) Architecture

**Figure 1:**
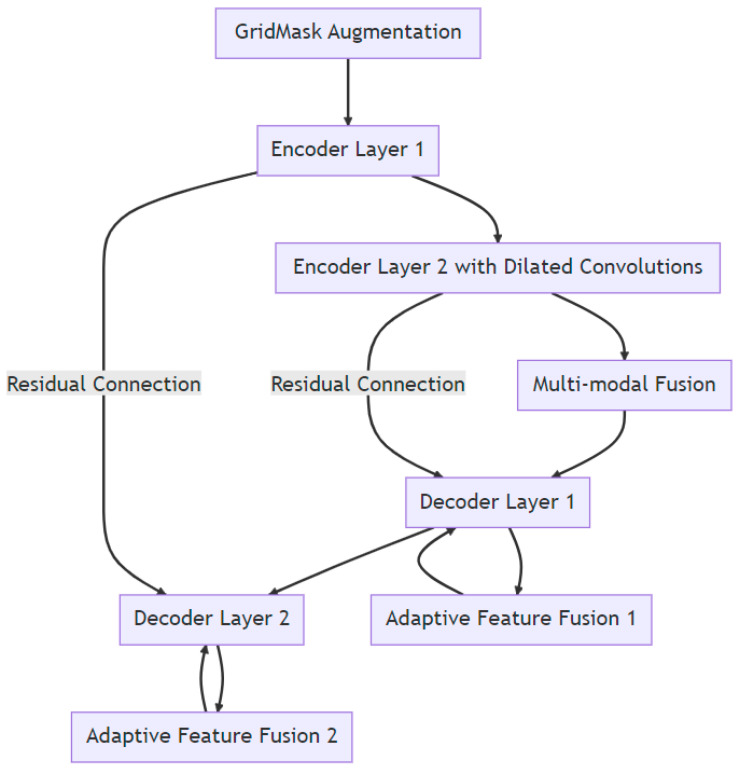
Visual representation of EUGNet.

To address the inherent challenges associated with the traditional U-Net architecture and to harness the potential benefits of the GridMask augmentation technique, we introduce the enhanced U-Net with GridMask (EUGNet) architecture. The following subsections detail the components and innovations of EUGNet:

1. Deep Contextual Encoder: To capture distant contextual information, which the traditional U-Net might miss, our encoder is deepened and incorporates dilated convolutions. This enhancement allows for a broader receptive field without a significant increase in computational complexity.
2. Residual Connections: To mitigate the loss of fine-grained spatial details during the downsampling and upsampling processes, we have integrated residual connections between corresponding encoder and decoder layers. This integration ensures the preservation of spatial information, aiding in more accurate segmentation output reconstruction.
3. Class Balancing Loss: Considering the frequent challenge of imbalanced class distributions in medical image analysis, our architecture employs a class-balancing loss function. This adjustment ensures that the model remains unbiased towards the majority class, providing equal importance to all classes during training.
4. Adaptive Feature Fusion: To better handle objects of irregular shapes, we introduce an adaptive feature fusion mechanism within the decoder. This mechanism adaptively weighs features from the encoder and the upsampled features from the preceding decoder layer, allowing the model to focus on the most pertinent features for segmentation.
5. GridMask Augmentation Module: The GridMask technique is directly integrated into our training pipeline. Before the images are input into the encoder, they undergo the GridMask module, ensuring the model consistently trains with augmented data, enhancing its robustness and reducing overfitting tendencies.
6. Efficient Implementation: To address U-Net’s computational demands, our architecture employs depthwise separable convolutions where feasible. This approach reduces the parameter count without compromising the model’s learning capacity.
7. Multi-Modal Fusion: For tasks that involve multiple data modalities, EUGNet introduces a fusion layer post-encoder. This layer is designed to effectively fuse features from different modalities before they are passed to the decoder. Figure 1 depicts a visual representation of EUGNet.

### 2.2. GridMask Algorithm

GridMask is a straightforward, versatile, and effective technique. When provided with an input image, our algorithm randomly eliminates certain pixels from it. In contrast to other approaches, our algorithm’s removal region is distinct in that it does not consist of continuous pixel clusters or randomly scattered pixels as in dropout. Instead, it removes a region made up of disconnected sets of pixels. The setting can be expressed as follows [53]:

(1) $$\tilde{X}=X\times M\;$$

where $X\in \;R^{H\times W\times C}$ represents the input image, $M\in \;{\left\{0,1\right\}}^{H\times W}$ is the binary mask that stores pixels to be removed, and $\tilde{X}\in \;R^{H\times W\times C}$ is the result produced by our algorithm. $R^{H\times W\times C}$ represents a 3-dimensional space for an image where *H* stands for the height of the image in pixels, *W* stands for the width of the image in pixels, and *C* stands for the number of channels in the image. For the binary mask *M*, if $M_{i,j}=1$, we keep pixel (i,j) in the input image; otherwise, it will be removed. The algorithm is applied after the image normalization operation.

**Figure 2:**
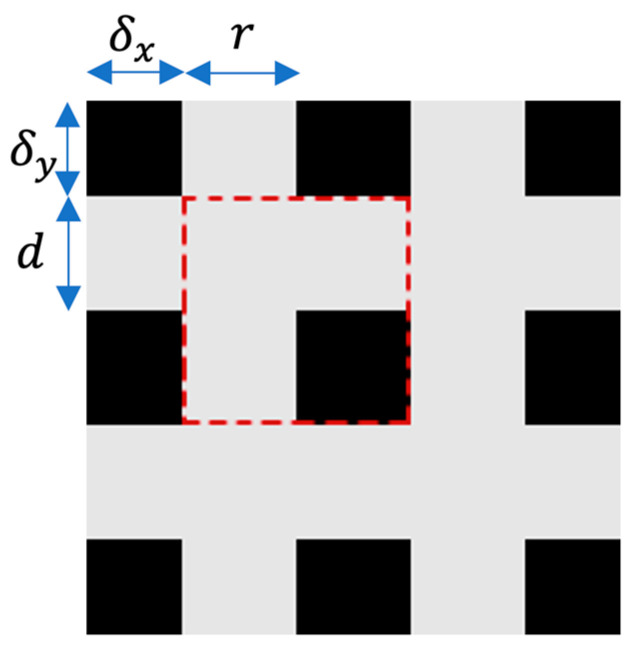
A single cell of the GridMask is illustrated by the red-dashed square.

**Figure 3:**
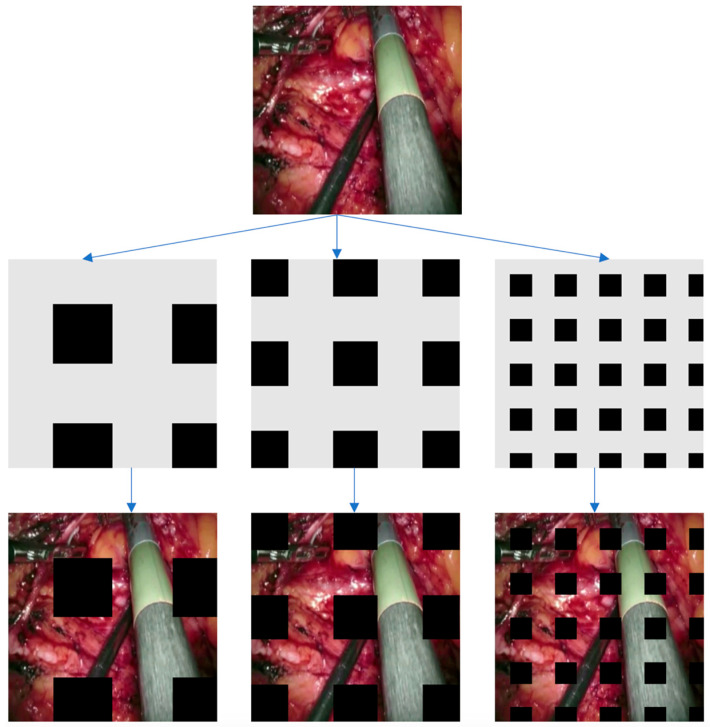
This illustration displays instances of GridMask in action. Initially, we generate a mask based on the specified parameters (*r*, *d*, *δ_x_*, *δ_y_*). Subsequently, we apply this mask to the input image through multiplication. The outcome is presented in the final image.

The shape of *M* looks like a grid, as shown in Figure 2. Four numbers $\left(r,d,\;\delta_{x},\;\delta_{y}\right)$ are used to represent a unique *M*. Every mask is formed by tiling the units, as shown in Figure 3. Here, *r* is the ratio of the shorter gray edge in a unit, and *d* is the length of one unit. $\delta_{x}$ and $\delta_{y}$ are the distances between the first intact unit and the boundary of the image.

### 2.3. Data Collection for Algorithm Evaluation

To show the robustness and generalization ability of the proposed framework for robotic instrument segmentation, a dataset with different robotic surgical scenarios and instruments has been used to validate the proposed architectures. This dataset consists of training and testing data for ex vivo robotic surgery with different articulated instruments:

(1) Da Vinci robotic (DVR) dataset [55]: The training set contains four ex vivo 45 s videos. The testing set consists of four 15 s and two 60 s videos. The test video features contain two instruments. Articulated motions are present in all the videos. The ground truth masks are automatically generated using joint encoder information and forward kinematics. Hand-eye calibration errors are manually corrected. All the videos have been recorded with the da Vinci Research Kit (dVRK) open-source platform [56]. The frames have a resolution of 720 × 576, and the videos run at 25 frames per second. This means that we have a total of (60 + 15) × 25 = 1875 frames (images).
(2) We obtained the recorded videos for testing our algorithm from open sources on the Internet, including the U.S. National Library of Medicine [57] (video links are available upon request). The videos showed various surgical procedures, such as midline lobectomy, right superior line lobectomy, thoracotomy, thoracoscopic lung surgery, and prostatectomy. Each video showed splash-like bleeding. The frames have a resolution of 720 × 576, and the videos run at 25 frames per second. The total duration of these videos is 2 min, which means 2 × 60 × 25 = 3000 frames (images).
(3) The binary segmentation EndoVis 17 [58] dataset, comprising 600 images, was utilized for both testing and training purposes. It consists of 10 sequences from abdominal porcine procedures recorded with da Vinci Xi systems. The dataset was curated by selecting active sequences that exhibited substantial instrument motion and visibility, with 300 frames sampled at a 1 Hz rate from each procedure. Frames where the instrument motion was absent for an extended period were manually excluded to maintain a consistent sequence of 300 frames. For the purpose of training, the first 225 frames from 8 sequences were made available, while the remaining 75 frames of these sequences were reserved for testing. Additionally, 2 sequences, each with a complete set of 300 frames, were exclusively allocated for testing.

### 2.4. Baseline Method and Evaluation Protocol

U-Net is a widely adopted tool in the field of medical image analysis, especially for segmenting surgical instruments in medical images and videos. To establish a baseline for comparison, we employ an advanced version of the U-Net architecture, known for its precision in segmenting robotic surgical tools. This choice is natural because the fine-grained U-Net architecture represents one of the cutting-edge convolutional models for this specific task. The frames are randomly chosen during training to present the networks with varying input data, as we are mostly interested in comparing the proposed architecture to the baseline method rather than achieving the highest scores, and GridMask data augmentation is performed. Because transfer learning carries the risk of transferring biases present in the source dataset to our target task, transfer learning is neglected. Since biases are undesirable for your application, training from scratch was preferred. In our experiments, the cyclical learning rate (CLR) bounds for the U-Net network are set to (10^−4^; 10^−2^. The quantitative metrics of choice to evaluate the predicted segmentations are mean Intersection over union (mean *IoU*) and mean Dice similarity coefficient (mean *DSC*):

(2) $$\bar{IoU}\left(\hat{y},y\right)=\frac{1}{K}\sum_{k=1}^{K}\frac{TP_{k}}{TP_{k}+FP_{k}+NF_{k}}\;$$

(3) $$\bar{DSC}\left(\hat{y},y\right)=\frac{1}{K}\sum_{k=1}^{K}\frac{2TP_{k}}{2TP_{k}+FP_{k}+NF_{k}}\;\;$$

where *K* = 2 (*k* = 0 background, *k* = 1 foreground), and $TP_{k}$, $FP_{k}$, and $NF_{k}$ represent true positives, false positives, and false negatives for class *k*, respectively.

All networks were trained and tested (including inference times) on a computer with a 13th generation Intel Core™ i9-13900KF processor (E-cores up to 4.30 GHz and P-cores up to 5.40 GHz) CPU and a NVIDIA GeForce RTX™ 4080 16GB GDDR6X GPU. The inference time was calculated, including data transfers from CPU to GPU and back, and averaged across 1000 inferences.

## 3. Results

Comparing the results of our proposed augmented data employing GridMask with the U-Net in the DVR testing set, we observed improvements of 1.6, 1.7, and 1.7 percentage points in balanced accuracy (foreground), mean intersection over union (IoU), and mean DSC, respectively (see Table 1). This has a significant impact on inference speed, which is reduced from 0.163 ms for the U-Net without GridMask to 0.097 ms or the U-Net with GridMask, becoming a viable real-time instrument–tissue segmentation method for robotic surgery with the da Vinci platform.

**Figure 4:**
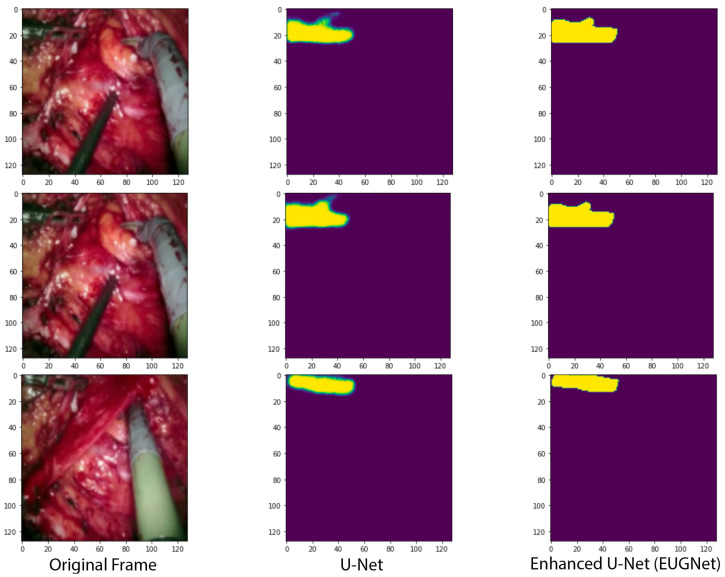
Qualitative comparison of our proposed convolutional architectures with GridMask data augmentation versus the state-of-the-art U-Net network.

The results of the U-Net with the GridMask method for data augmentation show an improvement over the U-Net without GridMask of 4.6, 2.9, and 6.2 percentage points in balanced accuracy (foreground), mean IoU, and mean DSC, respectively (see Table 1). The inference speed is also real-time, approximately 29 fps. The qualitative results in Figure 4 show how our proposed architecture respects the borders of the tooltip of left-handed surgical tools more.

**Figure 5:**
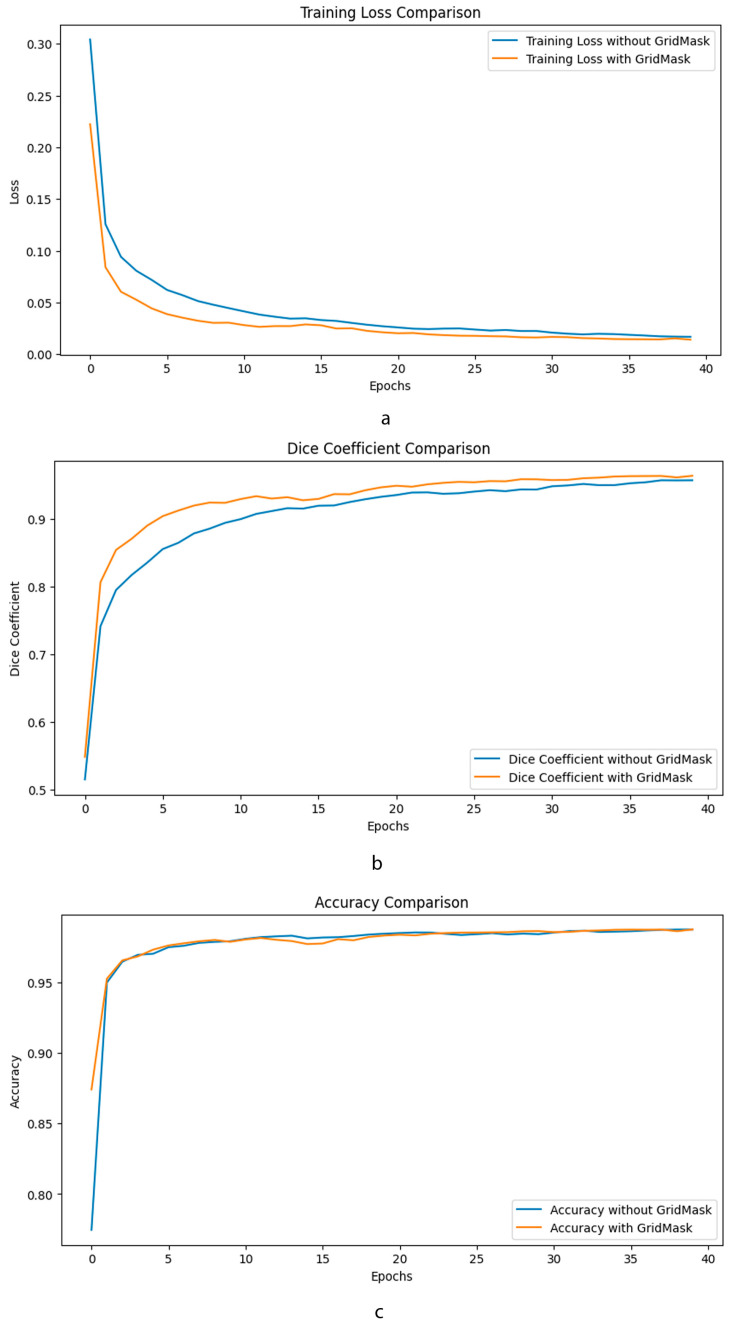
Comparative performance metrics of enhanced U-Net with GridMask data augmentation. This composite image showcases three key performance indicators—training loss (**a**), Dice coefficient (**b**), and accuracy (**c**)—over 40 epochs, comparing the outcomes of an enhanced U-Net.

As can be seen in Figure 5, the application of GridMask data augmentation in the training of an enhanced U-Net model has demonstrated notable improvements across various metrics. Specifically, the graphs provided illustrate that incorporating GridMask results in a more stable and generally lower training loss over 40 epochs, suggesting better generalization and less overfitting compared to training without GridMask. Accuracy metrics also show an improvement, with the model achieving comparable or slightly higher accuracy when trained with GridMask, implying that the model’s predictions are more reliable. Furthermore, the Dice coefficient, which is crucial for evaluating the model’s performance in segmentation tasks, shows a clear enhancement when GridMask is utilized. The model with GridMask maintains a consistently higher Dice coefficient, indicating superior overlap between the predicted and ground-truth segmentation masks. These results collectively suggest that the use of GridMask data augmentation can significantly bolster the performance of enhanced U-Net architectures in learning tasks.

## 4. Discussion

In the forthcoming stages of our research, we aim to refine our approach to using GridMask for data augmentation. Instead of uniformly applying GridMask to all training images, we intend to implement a more precise strategy. This refined approach involves overlaying masks exclusively onto the segmented regions of the images while excluding the background. By doing so, we aim to create a more targeted and effective data augmentation process. Furthermore, we plan to conduct an in-depth evaluation and comparison of the effectiveness of alternative augmentation techniques such as MixUp and CutMix. This comparative analysis will help us determine which augmentation strategy yields the most favorable results for our specific task. In our ongoing research, we will also explore the potential advantages of transfer learning, potentially harnessing the capabilities of deeper neural network architectures. Transfer learning involves leveraging pre-trained models to expedite training and potentially improve the performance of our model in surgical instrument segmentation.

The GridMask data augmentation technique led to performance improvement in the segmentation of surgical tools by introducing structured occlusion in training images. This encourages the U-Net model to learn more robust features by (a) forcing contextual learning: by partially occluding the surgical tools, the network must learn to infer the shape and position of tools from the visible context. (b) Improving generalization: GridMask helps in reducing overfitting by preventing the network from relying on specific visual cues that are only present in the training data. (c) Enhancing feature learning: it encourages the network to learn more comprehensive feature representations by not depending on any particular region of the tool, leading to a more versatile understanding of the tool’s appearance. (d) Simulating real-world occlusions: in a surgical environment, tools may be occluded by various objects, including human hands, tissues, or other tools.

By integrating these challenging scenarios during training, GridMask effectively enhances the robustness of the U-Net model for surgical tool segmentation tasks.

It should be emphasized that in the training of U-Net, standard data augmentation techniques are employed. These include rotation, flipping, scaling, translation, elastic deformation, brightness adjustment, noise injection, and cropping. The intent of our study is to evaluate the effectiveness of GridMask data augmentation in comparison to these widely used techniques. During the data preparation phase of training, we implement the aforementioned common augmentation strategies before proceeding to train the model with GridMask techniques. Therefore, our comparisons are not made against models without any data augmentation but against those that have been trained with the standard set of augmentation methods, excluding GridMask.

Additionally, we are considering the application of adversarial training as part of our research. This technique, which has demonstrated promising outcomes in broader semantic labeling applications, may play a role in further enhancing the precision and accuracy of our robotic surgical instrument segmentation model. One particularly captivating avenue for future investigation is the three-dimensional (3D) localization of segmented surgical instruments. With the growing prevalence of stereo endoscopes, we have the opportunity to segment both images produced by these devices. This dual-image segmentation enables us to align instrument pixels between the stereo images. By ensuring proper camera calibration, we can leverage these matched points for dense geometry-based triangulation, ultimately providing precise 3D localization information for surgical tools. This advancement could greatly enhance the spatial understanding and guidance of surgical procedures.

## 5. Conclusions

We have improved the current state-of-the-art U-Net architecture by employing GridMask data augmentation techniques. When we compare the outcomes of our novel data augmentation approach, which incorporates GridMask, with the U-Net model on the DVR testing dataset, we observe notable enhancements. Specifically, there are improvements of 1.6 percentage points in balanced accuracy for the foreground, 1.7 points in IoU, and 1.7 points in mean DSC. These improvements are highly significant and have a substantial impact on inference speed. The inference speed, which is a critical factor in real-time applications, has seen a noteworthy reduction. It decreased from 0.163 milliseconds for the U-Net without GridMask to 0.097 milliseconds for the U-Net with GridMask. This substantial improvement in inference speed positions our model as a viable real-time instrument–tissue segmentation method for robotic surgery, especially when deployed on the da Vinci platform.

Moreover, when we examine the results of the U-Net model with GridMask for data augmentation, we witness even more impressive performance gains compared to the U-Net with GridMask alone. There are remarkable improvements of 4.6 percentage points in balanced accuracy for the foreground, 2.9 points in mean IoU, and 6.2 points in mean DSC, as highlighted in Table 1. These improvements further underscore the efficacy of our approach.

Additionally, the inference speed remains in real time, operating at an approximate rate of 29 frames per second (fps). This fast processing rate is essential for the dynamic and time-sensitive nature of robotic surgical procedures.

## Figures and Tables

**Table 1:** Quantitative results for segmentation of non-rigid robotic instruments in testing set videos. IoU stands for intersection over union, and DSC for Dice similarity coefficient. The means are performed over classes, and the results presented are averaged across testing frames.

Network	Inference Time (ms/fps)	Balanced Accuracy (fg.)	Mean IoU	Mean DSC
U-Net without GridMask	62.1/16.1	82.5%	78.2%	84.2%
U-Net with GridMask	34.2/29.2	86.3%	80.6%	89.5%

## Data Availability

The data presented in this study are available at https://universe.roboflow.com/models/instance-segmentation (accessed on 12 September 2023) and https://www.ncbi.nlm.nih.gov/pmc/articles/PMC6462551/figure/vid/ (accessed on 14 August 2023).
